# Structural and molecular substrates underlying functional dysconnectivity and cognitive impairment in neuronal intranuclear inclusion disease

**DOI:** 10.3389/fnbeh.2026.1854155

**Published:** 2026-07-03

**Authors:** Shujun Zhang, Guorong Zhang, Yahui Ouyang, Guoping Wang, Yueqin Chen, Jiehuan Wang

**Affiliations:** 1 Department of Radiology, Affiliated Hospital of Jining Medical University, Jining, Shandong, China; 2 Department of Neurology, Affiliated Hospital of Jining Medical University, Jining, China; 3 College of Medical Imaging and Laboratory, Jining Medical University, Jining, Shandong, China

**Keywords:** cognition, functional connectivity strength, molecular substrates, NIID, white matter microstructure

## Abstract

**Background:**

Neuronal intranuclear inclusion disease (NIID) is a complex neurodegenerative disorder characterized by progressive cognitive decline. However, the patterns of functional network reorganization, as well as the underlying structural white matter (WM) damage and molecular mechanisms contributing to cognitive impairment, remain poorly understood.

**Methods:**

Nine patients with genetically-confirmed NIID (following the exclusion of one patient due to excessive head motion) and 10 age- and sex-matched healthy controls underwent multimodal MRI. We integrated voxel-level functional connectivity strength (FCS) with DTI-derived metrics (FA, AD, RD, and MD) across 20 major WM tracts. Cognitive performance was assessed via Montreal Cognitive Assessment (MoCA). We employed a “structure-function-metabolism” analytical pipeline to examine how WM disintegration relates to cortical dysconnectivity and how these changes align with normative receptor maps (via JuSpace) and cerebral blood flow.

**Results:**

NIID patients demonstrated a distinct pattern of functional reorganization, marked by decreased FCS in the right opercular part of the inferior frontal gyrus (IFGoperc) and increased FCS in the left gyrus rectus; notably, the reduced FCS in the right IFGoperc was positively correlated with MoCA scores. Significant WM degeneration was observed in the bilateral anterior thalamic radiation (ATR) and the forceps minor, with FA values in the latter showing a significant positive correlation with MoCA scores. Crucially, the severity of WM damage (reduced FA; elevated AD, RD, and MD) in the left ATR was significantly correlated with functional disconnection in the IFGoperc, suggesting a structural-functional cascade may underlie cognitive deterioration. Molecular enrichment analysis further demonstrated that these functional deficits were spatially coupled with specific serotonergic (5-HT1b/2a) and glutamatergic (mGluR5) receptor profiles, as well as regional hyperperfusion.

**Conclusion:**

Our findings suggest a potential multi-level pathophysiological framework in NIID, where structural disintegration of thalamo-cortical and interhemispheric tracts may serve as a topographical substrate for downstream functional network reorganization. This structural-functional-molecular axis, characterized by the convergence of network dysfunction, regional hyperperfusion, and specific neurotransmitter profiles, provides preliminary insights into the neurobiological mechanisms potentially underlying cognitive decline in NIID.

## Highlights

- NIID pathology manifests as a multi-dimensional disruption across structural, functional, and molecular networks.
- Topographical damage to thalamo-cortical and interhemispheric pathways may underlie cortical network dysfunction.
- Reduced right inferior frontal gyrus connectivity correlates with disease-specific cognitive decline.
- Neurotransmitter-weighted analysis links functional network deficits to specific molecular substrates.
- Regional hyperperfusion and functional reorganization define the unique metabolic-functional signature of NIID.

## 1 Introduction

Neuronal intranuclear inclusion disease (NIID) is a rare and heterogeneous multisystem neurodegenerative disorder characterized histopathologically by the presence of eosinophilic, ubiquitin-positive, and p62-positive intranuclear inclusions in the central and peripheral nervous systems, as well as in visceral organs (Sone et al., 2011, 2016; Takahashi-Fujigasaki, 2003). Clinically, NIID manifests with a broad spectrum of symptoms, including progressive cognitive decline, movement disorders, autonomic dysfunction, and paroxysmal encephalopathy episodes (Sone et al., 2011; Tai et al., 2023; Tian et al., 2022). For decades, definitive diagnosis relied solely on post-mortem examination or invasive skin biopsies. However, the diagnostic paradigm shifted significantly following the discovery of GGC repeat expansions in the non-coding region of the NOTCH2NLC gene as the primary genetic cause (Deng et al., 2019; Ishiura et al., 2019). Despite these diagnostic advances, the therapeutic options for NIID remain limited to symptomatic management, as the precise pathological mechanisms driving network-level brain dysfunction are not yet fully elucidated. Conventional neuroimaging plays a pivotal role in the clinical suspicion and longitudinal monitoring of NIID. Computed tomography often reveals non-specific cerebral atrophy or diffuse leukoencephalopathy in advanced stages. Magnetic resonance imaging (MRI) is more definitive, typically demonstrating symmetrical T2-weighted and fluid-attenuated inversion recovery (FLAIR) hyperintensities in the periventricular and deep white matter (WM) (Liu et al., 2023; Sugiyama et al., 2017; Suthiphosuwan et al., 2019; Zhu et al., 2024b). The hallmark radiological feature of NIID is the persistent high signal intensity on diffusion-weighted imaging (DWI) along the corticomedullary junction, which may precede the onset of clinical symptoms or widespread white matter lesions (Sone et al., 2016; Zhang et al., 2023c). Although the “DWI ribbon sign” is a key diagnostic feature, it does not adequately reflect the widespread changes in brain microstructure and functional connectivity, which show a close association with the patients’ progressive neurological symptoms.

In recent years, advanced neuroimaging techniques have begun to unravel the complex pathophysiology of NIID beyond routine sequences. Research into brain structural changes using diffusion tensor imaging (DTI) has consistently demonstrated widespread white matter damage. Metrics such as fractional anisotropy (FA), mean diffusivity (MD), axial diffusivity (AD), and radial diffusivity (RD) have revealed that NIID involves more than just localized corticomedullary lesions; it entails a global disruption of WM tract integrity, particularly in the anterior thalamic radiation and long association fibers (Luo et al., 2021; Lv et al., 2024; Zhu et al., 2024a). Concurrently, functional MRI (fMRI) studies have begun to document altered blood-oxygen-level-dependent (BOLD) signal dynamics, suggesting that the structural breakdown of WM pathways likely leads to secondary cortical dysfunction (Zhu et al., 2024a). However, the precise topographical relationship between these structural deficits and functional network organization remains insufficiently characterized.

A sophisticated approach to quantifying these functional alterations is the analysis of functional connectivity strength (FCS). FCS is a voxel-wise metric based on graph theory that calculates the average connectivity of a specific voxel with all other voxels in the brain (Buckner et al., 2009). Unlike seed-based analysis, which requires a priori selection of regions of interest, FCS provides a data-driven, whole-brain map of “functional hubs.” High FCS values represent brain regions that serve as critical nodes for information transfer (Zuo et al., 2011). In neurodegenerative diseases, FCS is highly sensitive to the breakdown of network hubs and can identify compensatory mechanisms or vulnerable nodes before significant atrophy occurs (Buckner et al., 2009). In NIID, evaluating FCS allows for a comprehensive assessment of how the disease reshapes the functional landscape of the brain.

Despite the clinical importance of cognitive decline in NIID, previous neuroimaging studies have primarily focused on isolated modalities, such as either microstructural WM damage using DTI (Lv et al., 2024) or localized functional connectivity changes (Zhu et al., 2024a). To date, no study has integrated functional connectivity metrics with microstructural WM integrity and normative molecular receptor maps in NIID. The pathological progression of NIID is thought to trigger a cascade of network Failures. Structural disruptions in thalamo-cortical and callosal pathways likely manifest as functional connectivity deficits in distributed cortical regions, potentially correlating with the executive dysfunction and memory impairment measured by the Montreal Cognitive Assessment (MoCA) (Lv et al., 2024; Sone et al., 2016; Zhu et al., 2024a). Furthermore, the neurochemical basis for these network disruptions in NIID remains an unexplored frontier. While other neurodegenerative diseases like Alzheimer’s or Parkinson’s have well-mapped neurotransmitter imbalances, the molecular environment of the NIID brain (specifically regarding serotonergic, GABAergic, and glutamatergic systems) remains poorly understood. Mapping these functional deficits onto normative neurotransmitter receptor atlases (such as via JuSpace analysis) could bridge the gap between macroscopic network failure and microscopic metabolic dysfunction (Dukart et al., 2020).

Therefore, this study aims to provide a multimodal characterization of NIID by integrating DTI-based WM tractography with resting-state FCS mapping. We seek to: (1) identify core functional hubs that are disrupted in NIID; (2) determine whether these functional deficits are spatially and statistically linked to specific white matter fiber tract damage; (3) evaluate the clinical relevance of these changes in relation to cognitive scores; and (4) explore the potential neurotransmitter landscapes and cerebral blood flow (CBF) patterns that co-localize with functional abnormalities. By establishing this structural-functional-molecular framework, we hope to identify novel imaging biomarkers and potential pharmacological targets to address the complex pathology of NIID.

## 2 Materials and methods

### 2.1 Participants

A total of 10 adult patients with NIID were prospectively enrolled from the Affiliated Hospital of Jining Medical University between January 2023 and June 2024. The inclusion criteria for NIID patients were: (1) age ≥ 18 years; (2) definitive diagnosis via skin biopsy and/or genetic testing confirming NOTCH2NLC GGC repeat expansion; and (3) clinical stability with the willingness and ability to undergo MRI procedures. Participants were excluded if they met any of the following criteria: (1) neuroimaging evidence of cerebral infarction, lacunar infarcts, or cerebral hemorrhage, or a history of other neurological or psychiatric disorders; (2) leukoencephalopathy secondary to demyelinating diseases, toxin exposure, infection, or neoplasms; (3) standard contraindications to MRI (e.g., pacemakers or metallic implants). Additionally, 10 age- and sex-matched healthy controls (HCs) were recruited. This study was approved by the Medical Ethics Committee of the Affiliated Hospital of Jining Medical University. All participants or their legal guardians provided written informed consent prior to enrollment. The study complied with the Declaration of Helsinki and local regulations concerning human subject’s research.

### 2.2 Neuropsychological assessment

Global cognitive function was independently assessed by at least two board-certified senior neurologists (each with >10 years of clinical experience) who were blinded to participants’ clinical and genetic information. Cognitive status was measured using the Montreal Cognitive Assessment (MoCA) (Nasreddine et al., 2005), administered according to standardized procedures in a quiet clinical setting. The MoCA provides a comprehensive evaluation of cognitive function, encompassing executive function, visuospatial skills, naming, attention, abstraction, and delayed recall. This widely utilized screening tool is scored from 0 to 30, with lower scores reflecting greater cognitive impairment.

### 2.3 Image acquisition

All neuroimaging data were acquired on a 3.0-Tesla MAGNETOM Prisma MRI scanner (Siemens Healthineers, Erlangen, Germany) equipped with a 24-channel phased-array head coil. To attenuate acoustic noise and minimize head motion, all participants wore earplugs and were positioned with tight yet comfortable foam padding. The imaging protocol included high-resolution 3D T1-weighted imaging (3D-T1WI), resting-state functional MRI (rs-fMRI), and DTI. High-resolution structural images were captured using a 3D T1-weighted magnetization-prepared rapid gradient-echo (MPRAGE) sequence with the following parameters: repetition time (TR) = 2300ms; echo time (TE) = 3.2 ms; inversion time (TI) = 900 ms; flip angle (FA) = 12°; field of view (FOV) = 256 mm × 256 mm; matrix size = 256 × 256; slice thickness = 1 mm (no gap); and 167 sagittal slices. Functional data were collected using a gradient-echo single-shot echo-planar imaging (GRE-SS-EPI) sequence: TR = 1,500 ms; TE = 30 ms; FA = 90°; FOV = 220 mm × 220 mm; matrix size = 64 × 64; slice thickness = 2 mm with a 1-mm gap; 72 axial slices acquired in an interleaved order; 240 volumes; and a total acquisition time of 360 s. During the rs-fMRI scan, participants were instructed to remain still, keep their eyes closed, and avoid thinking of anything in particular. Diffusion-weighted data were acquired using a spin-echo single-shot echo-planar imaging (SE-SS-EPI) sequence: TR = 10,000 ms; TE = 74 ms; FA = 90°; FOV = 256 mm × 256 mm; matrix = 128 × 128; slice thickness = 3 mm (no gap); and 68 axial slices. Diffusion sensitization was applied along 64 non-collinear directions (b = 1,000 s/mm^2^) and five additional b = 0 s/mm^2^ images, with a total acquisition time of 700 s.

### 2.4 Functional MRI data analysis

Preprocessing of the resting-state BOLD data was conducted using SPM12 and the Data Processing and Analysis for Brain Imaging (DPABI^1^) toolbox (Yan et al., 2016), following established protocols (Zhang et al., 2022, 2023a,2023b). To ensure longitudinal magnetization stability and allow for participant acclimation to the scanning environment, the initial 10 volumes of each session were discarded. The remaining dynamics underwent slice-timing correction and subsequent realignment to mitigate inter-volume head motion. Participants were included only if their maximum head displacement was <2.0 mm and rotational motion was <2.0° in any direction. Consequently, one patient with NIID was excluded from the study due to excessive head motion. This resulted in a final analytical sample of nine NIID patients and 10 healthy controls. Additionally, framewise displacement (FD) was calculated to assess volume-to-volume changes in head position. To further suppress physiological noise and motion artifacts, several nuisance covariates were regressed from the time series, including the Friston-24 motion parameters, white matter and cerebrospinal fluid signals, and linear trends. Motion-contaminated “spike” volumes (FD > 0.5 mm) were also identified and regressed out. Consistent with ongoing methodological discussions regarding the induction of artificial anti-correlations, global signal regression was not performed. The data were subsequently band-pass filtered (0.01–0.1 Hz) to isolate low-frequency fluctuations. For spatial normalization, individual structural scans were first co-registered with the mean functional image and then segmented. These were then transformed into the Montreal Neurological Institute (MNI) space using the DARTEL technique (Ashburner, 2007). Finally, the filtered functional images were warped to the MNI template using the resulting deformation fields and resampled to 3-mm isotropic voxels.

Functional Connectivity Strength (FCS) is a graph-theoretical metric representing a voxel-wise measure of weighted degree centrality, which quantifies the intrinsic functional importance of each voxel by assessing its connectivity with all other voxels within the gray matter mask (Buckner et al., 2009; Tomasi and Volkow, 2010). By identifying “functional hubs” through unbiased whole-brain mapping without the constraints of a-priori seed selection, FCS is particularly well-suited for characterizing the complex and potentially heterogeneous network failures in NIID, offering a more comprehensive perspective than traditional seed-based or ICA-derived approaches. For each participant, a voxel-wise connectivity matrix was derived by calculating the Pearson correlation coefficients between the BOLD time series of all pairs of voxels. To minimize the influence of spurious correlations resulting from physiological noise, the FCS was defined as the sum of positive correlation coefficients exceeding a predetermined threshold of *r* > 0.25. Subsequently, the individual FCS maps were standardized by dividing the value of each voxel by the global mean FCS to enhance inter-subject comparability. As a final step, the normalized FCS maps were spatially smoothed using a 6-mm full-width at half-maximum (FWHM) Gaussian kernel.

### 2.5 Structural MRI data analysis

Diffusion tensor imaging data preprocessing was performed using the Pipeline for Analyzing brain Diffusion images (PANDA^2^) (Cui et al., 2013), which integrates the FMRIB Software Library (FSL^3^) (Smith et al., 2004) and the Diffusion Toolkit (DTK^4^). Briefly, the diffusion-weighted images were first registered to the first b_0_ volume using an affine transformation to correct for eddy current-induced distortions and head motion. After skull-stripping, the diffusion tensor was calculated to estimate six independent components. Whole-brain deterministic fiber tractography was then conducted using the Fiber Assignment by Continuous Tracking (FACT) algorithm (Becher et al., 1999), with a fractional anisotropy (FA) threshold of 0.2 and a maximum curvature angle of 45°. To ensure anatomical precision, individual FA images were non-linearly registered to the MNI space. The resulting inverse transformation was then applied to warp the JHU White-Matter Tractography Atlas from the MNI space back to each participant’s native space. Based on this atlas-based approach, 20 primary white matter tracts were automatically segmented, and diffusion metrics were extracted from each tract for further analysis (Hua et al., 2008; Wakana et al., 2007).

### 2.6 Statistical analysis

Demographic and behavioral data were analyzed using IBM SPSS Statistics version 26.0 (SPSS Inc., Chicago, IL, United States). We implemented a multi-stage analytical framework to investigate FCS, white matter (WM) microstructural integrity, cognitive impairment, and their potential molecular underpinnings in patients with NIID. Sex was treated as a categorical variable (male = 1, female = 2). First, to identify functional alterations, voxel-wise comparisons of FCS maps between the NIID and HC groups were performed using two-sample *t*-tests in SPM12^5^. This analysis was restricted to a gray matter mask, with age and sex included as covariates. Multiple comparisons were corrected using a cluster-level family-wise error (FWE) method (cluster-defining threshold: *p* < 0.001; corrected significance: *p* < 0.05). Second, structural damage was assessed by comparing diffusion metrics (FA, AD, RD, and MD) across 20 major white matter tracts between the two groups. To account for multiple comparisons across these tracts, a Bonferroni correction was applied, setting the significance threshold at *p* < 0.0025 (0.05/20) for each DTI metric analyzed independently. Third, to integrate functional, structural, and clinical findings, mean FCS values were extracted from the ROIs exhibiting significant group differences. Pearson correlation analyses were then conducted within the NIID group to evaluate the interrelationships between these abnormal FCS values, indices of WM tract degeneration, and cognitive performance (e.g., MoCA scores). Finally, to elucidate the neurochemical basis underlying the observed functional deficits, we performed spatial correlation analyses between the group-level t-statistic map (NIID vs. HC) and normative PET-derived neurotransmitter atlases using the JuSpace toolbox (Dukart et al., 2020). This analysis covered multiple neurotransmitter systems, including dopamine, serotonin (5-HT), glutamate, chemoarchitecture, acetylcholine, opioids, cannabinoids, norepinephrine, and fludopa (Aghourian et al., 2017; Alakurtti et al., 2015; Beliveau et al., 2017; Fan et al., 2016; Kaller et al., 2017; Nørgaard et al., 2021; Normandin et al., 2015; Savli et al., 2012; Xu et al., 2024). Specifically, Spearman correlation coefficients were calculated between the t-map and neurotransmitter maps across 210 cortical regions defined by the Brainnetome atlas. These correlations were corrected for spatial autocorrelation and partial volume effects (PVE) based on gray matter probability maps. The exact *p*-values were determined via 5,000 spatial permutation tests (spin-tests). Results were considered statistically significant at a Bonferroni-corrected level of *p* < 0.05.

## 3 Results

### 3.1 Demographic and clinical features

A total of nine patients with NIID (four males and five females; mean age 58.00 ± 13.60 years) were enrolled, consisting of three cases with acute onset and six with chronic onset. The mean MoCA scores for the NIID group were 21.67 ± 4.27. Ten healthy controls (HCs) were included (five males and five females; mean age 56.56 ± 11.97 years). No significant differences were observed between the two groups in gender (*t* = 0.06, *p* = 0.81) or age (*t* = 0.24, *p* = 0.82).

### 3.2 Intergroup differences of FCS

**FIGURE 1:**
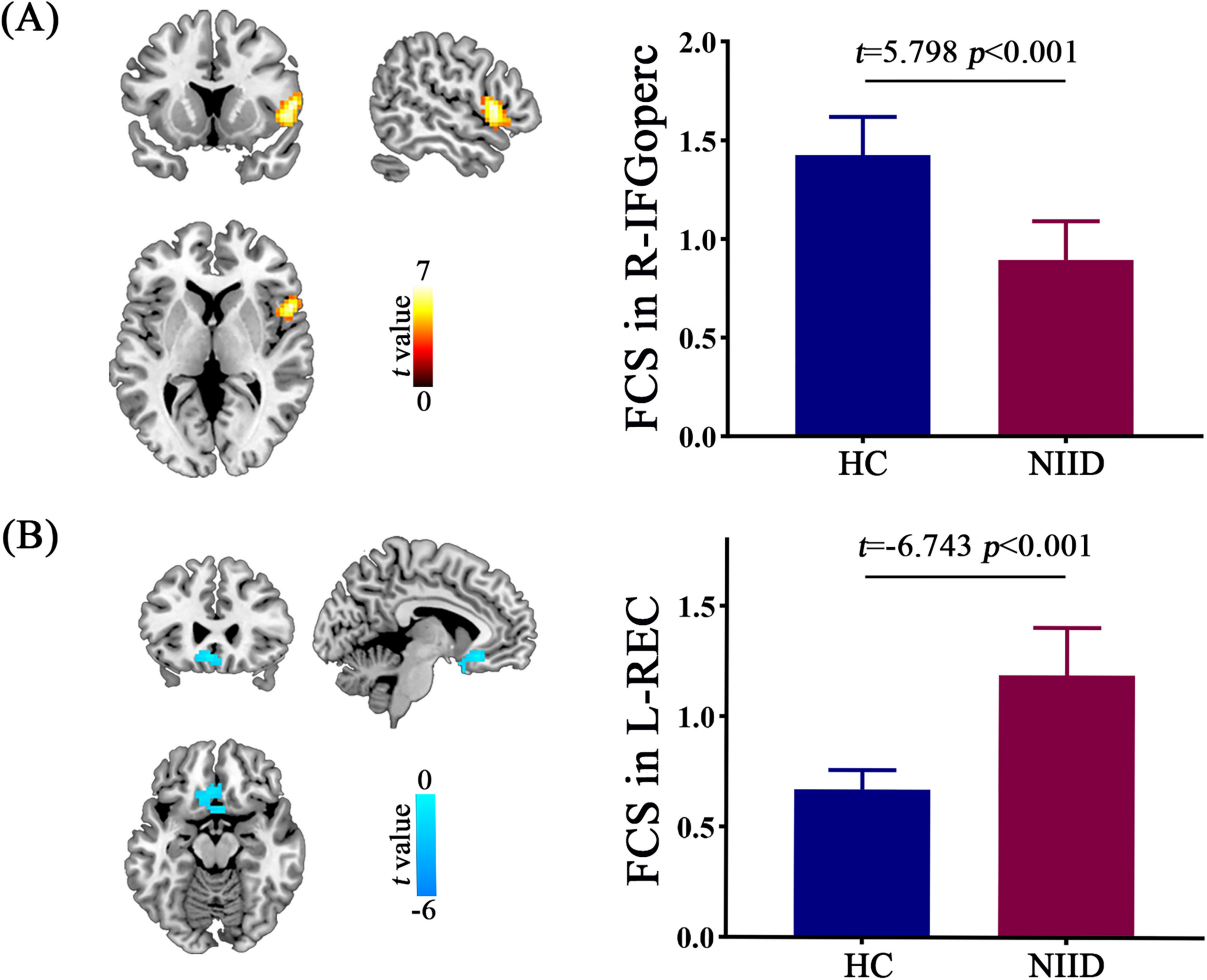
Differences in functional connectivity strength (FCS) between patients with neuronal intranuclear inclusion disease (NIID) and healthy controls (HC). **(A)** Brain maps and bar charts illustrate regions showing significantly decreased FCS in the NIID group, primarily in the right inferior frontal gyrus, pars opercularis (R-IFGoperc). **(B)** Brain maps and bar charts illustrate regions showing significantly increased FCS in the NIID group, such as the left rectus gyrus (L-REC). The statistical t-maps represent the contrast of (HC minus NIID). Warm colors (yellow-red) indicate regions where FCS is higher in the HC group (HC > NIID), while cold colors (blue-cyan) indicate regions where FCS is lower in the HC group (HC < NIID). Statistical significance was determined using two-sample *t*-tests, with multiple comparisons corrected using the False Discovery Rate (FDR) method at a cluster-level of *p* < 0.05 (following an initial voxel-level threshold of *p* < 0.001). Error bars in the bar graphs represent the standard deviation. NIID, neuronal intranuclear inclusion disease; HC, healthy control; FCS, functional connectivity strength; R-IFGoperc, right inferior frontal gyrus (pars opercularis); L-REC, left rectus gyrus; L, left; R, right.

Compared to the HC group, the NIID group exhibited significantly lower FCS in the right opercular part of the inferior frontal gyrus (IFGoperc: cluster size = 141 voxels, peak MNI coordinate x/y/z = 54/12/6, peak *t* = 7.45; *p* < 0.05, cluster-level FWE corrected; Figure 1A), and higher FCS in the left gyrus rectus (REC: cluster size = 113 voxels, peak MNI coordinate x/y/z = −12/21/−12, peak *t* = 6.29; *p* < 0.05, cluster-level FWE corrected; Figure 1B). To corroborate the voxel-wise findings, post-hoc ROI-based analyses were performed by extracting the average FCS values from the right IFGoperc and the left gyrus rectus. Independent-sample *t*-tests confirmed a significant decrease in the right IFGoperc FCS (*t* = 5.798, *p* < 0.001) and a significant increase in the left gyrus rectus FCS (*t* = −6.743, *p* < 0.001) in the NIID group compared to HCs. To evaluate the robustness of our results despite the limited cohort, we performed a post-hoc power analysis. For voxel-wise FCS, the effect sizes in the right IFGoperc and left REC were remarkably large (Cohen’s *d* = 2.73 and 3.18, respectively), yielding a post-hoc statistical power exceeding 99% (α = 0.05, two-tailed).

### 3.3 Intergroup differences of WM microstructural integrity

**FIGURE 2:**
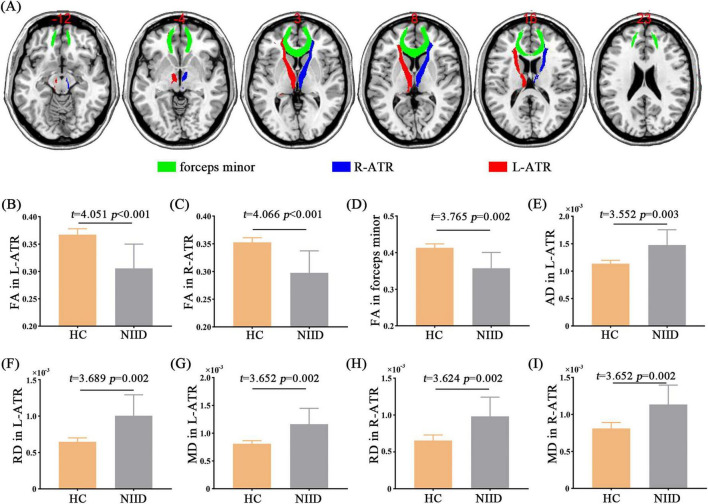
White matter tracts showing significant alterations in neuronal intranuclear inclusion disease (NIID) patients. **(A)** Visualization of the affected white matter tracts on representative axial slices. For enhanced clarity, each tract is directly labeled on the panel with its name printed in the corresponding color [Green: forceps minor; Blue: right anterior thalamic radiation (R-ATR); Red: left anterior thalamic radiation (L-ATR)]. **(B–I)** Quantitative comparisons of DTI metrics between the NIID and healthy control (HC) groups, including fractional anisotropy (FA), axial diffusivity (AD), radial diffusivity (RD), and mean diffusivity (MD) within the identified tracts. Data are presented as mean ± standard deviation. *t* and *p* values are indicated above the bars. L-ATR, left anterior thalamic radiation; R-ATR, right anterior thalamic radiation.

The anatomical distribution of the bilateral anterior thalamic radiation (ATR) and the forceps minor is illustrated in Figure 2A. Compared with the HC group, patients with NIID exhibited significantly lower FA values in the bilateral ATR and the forceps minor (all *p* < 0.05, Bonferroni corrected; Figures 2B–D). Regarding other diffusion metrics, significantly increased AD, RD, and MD values were observed in the left ATR (Figures 2E–G), while significantly higher RD and MD values were found in the right ATR of the NIID group (all *p* < 0.05, Bonferroni corrected; Figures 2H, I). For the eight white matter tracts showing significant differences, Cohen’s d values ranged from 1.63 to 1.87, with all post-hoc power values remaining above 90% (see Supplementary Table 1). These results suggest that the pathophysiological alterations in NIID provide a very strong signal that remains detectable even in a small group.

### 3.4 Correlations among FCS, WM integrity, and cognitive performance

**FIGURE 3:**
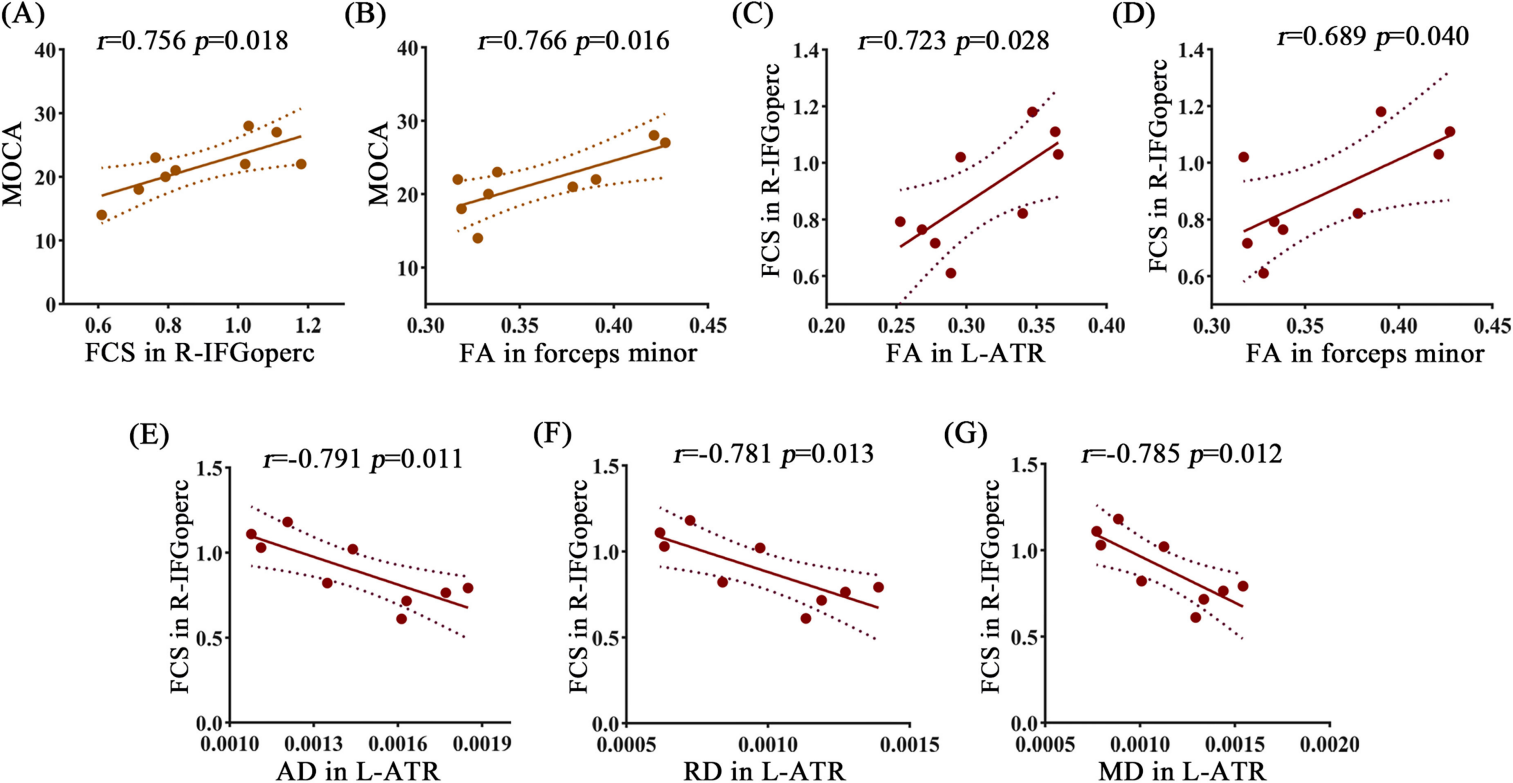
Correlations between clinical cognitive scores, functional connectivity, and white matter integrity. **(A,B)** Positive correlations between Montreal Cognitive Assessment (MoCA) scores and imaging metrics (FCS in R-IFGoperc and FA in forceps minor). Lower MoCA scores reflect greater cognitive impairment; thus, these positive correlations indicate that more severe cognitive deficit is associated with reduced functional connectivity and impaired white matter integrity in these regions. **(C–G)** Correlations between functional connectivity strength (FCS in R-IFGoperc) and structural DTI metrics (FA, AD, RD, and MD) across affected white matter tracts. Specifically, FCS shows positive correlations with FA **(C,D)** and negative correlations with AD, RD, and MD **(E–G)** in the L-ATR and forceps minor. The solid lines represent the linear regression, and the shaded areas indicate the 95% confidence intervals. *r* represents the Pearson correlation coefficient. MoCA, Montreal Cognitive Assessment; FCS, functional connectivity strength; R-IFGoperc, right inferior frontal gyrus (pars opercularis); L-ATR, left anterior thalamic radiation; FA, fractional anisotropy; AD, axial diffusivity; RD, radial diffusivity; MD, mean diffusivity.

Correlation analyses within the NIID group revealed significant associations between multi-modal imaging metrics and clinical cognitive performance. Specifically, FCS in the right IFGoperc was positively correlated with MoCA scores (*r* = 0.756, *p* = 0.018; Figure 3A). Similarly, FA values in the forceps minor exhibited a significant positive correlation with MoCA scores (*r* = 0.766, *p* = 0.016; Figure 3B).

Furthermore, we observed significant structure-function coupling between WM microstructural integrity and FCS. FCS in the right IFGoperc was positively correlated with FA values in both the left ATR (*r* = 0.723, *p* = 0.028; Figure 3C) and the forceps minor (*r* = 0.689, *p* = 0.040; Figure 3D). In contrast, diffusivity metrics in the left ATR, including AD (*r* = −0.791, *p* = 0.011; Figure 3E), RD (*r* = −0.781, *p* = 0.013; Figure 3F), and MD (*r* = −0.785, *p* = 0.012; Figure 3G), were all significantly and negatively associated with FCS in the right IFGoperc.

To account for the small sample size (*N* = 9) and minimize the influence of potential outliers, Spearman’s rank correlation (ρ) was employed to evaluate the associations between clinical variables and imaging metrics. These non-parametric analyses corroborated our initial findings, with primary associations remaining significant. Furthermore, we performed non-parametric bootstrapping (1,000 iterations) to calculate 95% Bias-Corrected and accelerated (BCa) confidence intervals (CIs). The correlations between FCS in the right IFGoperc and left ATR metrics (including FA, MD, AD, and RD) were confirmed to be robust, as their 95% BCa CIs did not encompass zero. However, none of these correlations survived the Benjamini-Hochberg FDR correction for multiple comparisons. Consequently, these results are considered exploratory and should be interpreted with caution. Detailed results of these correlation analyses are provided in the Supplementary Table 2.

### 3.5 Neurochemical underpinnings of FCS alterations

**FIGURE 4:**
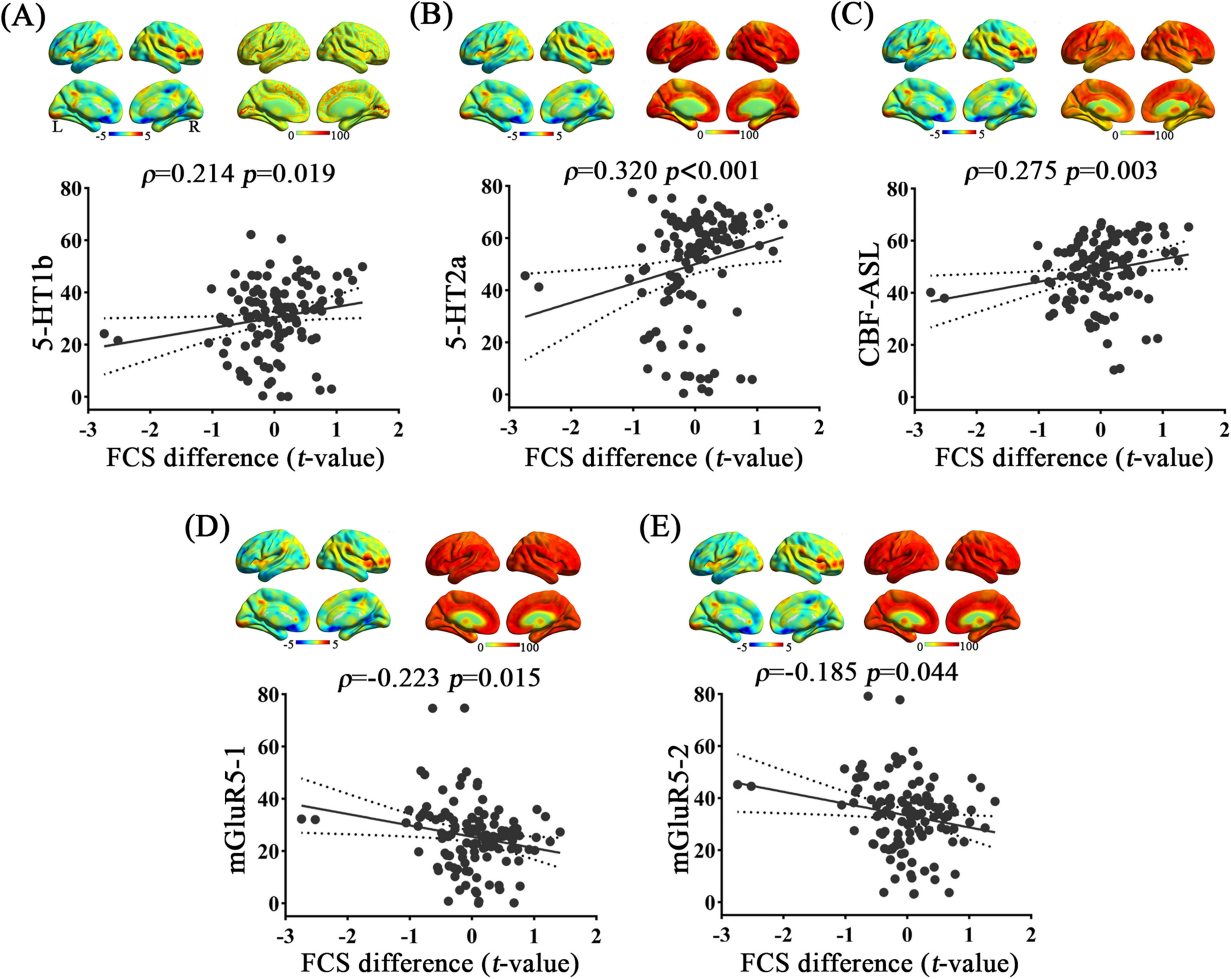
Spatial association between functional connectivity alterations and the distributions of neurotransmitter receptors and cerebral blood flow. **(A–E)** Spatial correlations between the group-level FCS alterations (*t*-map of HC minus NIID) and five normative neurobiological maps: serotonin receptors (5-HT1b, 5-HT2a), cerebral blood flow (CBF-ASL) and metabotropic glutamate receptors (mGluR5, shown in two representative maps). In each panel, the top row displays the brain surface representations used for the analysis: the statistical *t*-map of FCS differences (left) and the corresponding neurotransmitter density or blood flow map (right). The scatterplots illustrate the spatial correlation between these maps, where each data point represents one of the 210 cortical regions from the Brainnetome atlas. Solid lines represent the linear regression, and dashed lines indicate the 95% confidence intervals. Statistical significance was confirmed using spin tests to account for spatial autocorrelation (*p*_*spin*_ < 0.05). FCS, functional connectivity strength; HC, healthy control; NIID, neuronal intranuclear inclusion disease; CBF, cerebral blood flow; ASL, arterial spin labeling; 5-HT, serotonin; mGluR5, metabotropic glutamate receptor 5.

As illustrated in Figure 4, cross-region spatial correlation analyses using Spearman’s rank correlation revealed that the spatial topography of FCS alterations in patients with NIID was significantly associated with specific neurotransmitter systems (permutation-based *p* < 0.05). Specifically, the NIID-related FCS t-map exhibited significant positive spatial correlations with the serotonin system (5-HT1b: ρ = 0.214, *p* = 0.019; 5-HT2a: ρ = 0.320, *p* < 0.001) and cerebral blood flow (CBF-ASL: ρ = 0.275, *p* = 0.003). Additionally, significant negative spatial correlations were maintained with the metabotropic glutamate receptor 5 (mGluR5-1: ρ = -0.223, *p* = 0.015; mGluR5-2: ρ = −0.185, *p* = 0.044).

## 4 Discussion

The present study provides a comprehensive characterization of the structural and molecular substrates underlying cognitive impairment in NIID. By integrating voxel-based FCS, diffusion-based tractography, and normative neurotransmitter maps, our findings suggest that NIID is a multifaceted “disconnection syndrome.” Specifically, the breakdown of key white matter tracts, such as the ATR and forceps minor, serves as the structural foundation for the functional decoupling of frontal hubs, while the spatial distribution of this dysfunction is governed by the brain’s underlying chemoarchitecture, particularly the serotonergic systems.

Our results revealed a significant reduction of FCS in the IFGoperc, which was positively correlated with MoCA scores. The IFGoperc is a critical node in the executive control and salience networks, essential for inhibitory control and cognitive flexibility (Aron et al., 2014). Similar patterns of structural and functional disintegration in this region have been robustly associated with executive deficits in other neurodegenerative conditions, including frontotemporal dementia and Alzheimer’s disease (Schroeter et al., 2006). In the context of NIID, the characteristic eosinophilic intranuclear inclusions and extensive leukoencephalopathy appear to preferentially target the prefrontal cortex and its underlying subcortical architecture (Sone et al., 2016; Zhu et al., 2024a,b). This focal FCS reduction may reflect a “deafferentation” process, whereby the cortical node loses its communicative efficiency as its subcortical inputs are progressively severed. Meanwhile, the heightened FCS in the left gyrus rectus likely signifies a compensatory, albeit potentially maladaptive, reorganization. While this pattern aligns with the scaffolding theory of aging and cognition (STAC) suggesting the recruitment of alternative neural circuits to mitigate structural decline (Goh and Park, 2009; Park and Reuter-Lorenz, 2009; Reuter-Lorenz and Park, 2014), the correlation between IFGoperc integrity and MoCA scores indicates that the lateral prefrontal nodes remain the primary determinant of cognitive status in NIID.

Neuronal intranuclear inclusion disease is clinically characterized by high-intensity signals at the corticomedullary junction on DWI and progressive white matter rarefaction (Zhang et al., 2023c). Our DTI findings corroborate and extend these observations, identifying the bilateral ATR and forceps minor as the primary sites of microstructural degeneration, consistent with previous literature (Luo et al., 2021; Lv et al., 2024; Zhu et al., 2024a). Our results further elucidate a structural-functional cascade in NIID: the structural integrity of the ATR, a vital component of the limbic-thalamo-cortical circuit, directly influences downstream cortical function. Supported by the Multimodal White Matter Atlas (MWMA), which maps white matter microstructural tracts to functional network topology (Zhou et al., 2026), we observed that the severity of left ATR damage was significantly associated with functional dysconnectivity in the right IFGoperc. This suggests that structural tract degeneration in NIID directly disrupts corresponding cortical network hubs. Pathologically, elevated RD and MD in the ATR serve as sensitive markers for myelin loss, whereas alterations in AD typically reflect axonal fragmentation (Assaf and Pasternak, 2007; Mori et al., 2009; Song et al., 2002, 2003). Given that NIID is associated with *NOTCH2NLC* GGC repeat expansions (Sone et al., 2019), it is plausible that oligodendrocyte dysfunction leads to compromised myelin integrity and axonal conduction fidelity (Fields, 2015), subsequently disrupting neuronal synchrony and manifesting as the functional dysconnectivity detected here (Pajevic et al., 2014; Zhu et al., 2024a). Finally, the positive correlation between forceps minor FA and MoCA scores reinforces the critical role of interhemispheric connectivity in cognitive preservation. Collectively, our results indicate that thalamo-frontal tract disruption potentially underlies downstream cortical network dysfunction and progressive clinical deterioration in patients with NIID. However, these associations require future confirmation through direct clinicopathological evidence.

The application of JuSpace allowed us to transcend macroscale imaging and probe the neurochemical correlates of NIID. The significant spatial correlation between FCS t-maps and 5-HT1b/2a receptor distributions is highly revealing. The serotonergic system is known to modulate synaptic plasticity and executive function (Schmitt et al., 2006; Seymour et al., 2012). The overlap between functional decline and 5-HT receptor-rich regions suggests that these neurotransmitter systems may be particularly vulnerable to the metabolic stress induced by proteinaceous inclusions (Kish et al., 1985). The negative correlation with mGluR5 distribution offers a novel perspective. mGluR5 is involved in glutamatergic signaling and neuroinflammation. The finding that regions with lower mGluR5 density exhibit greater FCS loss may indicate a regional exhaustion of excitatory synaptic density or a failure of the glutamatergic system to sustain metabolic demands in the advanced stages of the disease. Furthermore, the positive correlation with CBF maps indicates that the most metabolically active regions, characterized by higher perfusion requirements, are the most susceptible to functional degradation, thereby supporting the “metabolic cost” hypothesis of neurodegeneration in NIID (Tai et al., 2022).

The interpretation of our findings should be considered in light of several limitations. First, our sample size is relatively small, which is a direct consequence of the ultra-rare nature of NIID and the rigorous requirements for genetic confirmation and MRI data quality. While post-hoc power analyses demonstrated robust effect sizes (Cohen’s *d* > 1.5, power > 85%) for our primary findings, the modest cohort size may still limit statistical power and the generalizability of the results. Therefore, these findings should be considered preliminary. Future multi-center collaborations with larger cohorts are essential to validate these structural-functional-molecular associations and further characterize the pathophysiology of NIID. Second, our correlation analyses were exploratory; thus, we did not apply multiple comparison corrections to minimize the risk of Type II errors and avoid obscuring potentially meaningful biological signals. This approach was intended to identify candidate neuroimaging markers for further investigation in NIID. Finally, the absence of systematic cognitive assessments in the healthy control group precluded a direct cross-group comparison of brain-behavior associations. Future studies incorporating comprehensive cognitive profiling across both cohorts are warranted to validate these preliminary findings.

Collectively, these findings suggest that cognitive decline in NIID may be characterized as a system-wide network dysfunction rather than a localized cortical phenomenon. The *NOTCH2NLC* expansion is potentially associated with progressive myelin degradation, as indicated by structural alterations in the anterior thalamic radiation and forceps minor. Such damage may subsequently compromise the functional integrity of key frontal hubs, including the pars opercularis of the inferior frontal gyrus. Furthermore, this topographical distribution appears to align with specific molecular gradients, demonstrating a spatial correlation with regions of high serotonergic receptor densities. While these observations offer potential neuroimaging markers for monitoring disease progression, the efficacy of therapeutic strategies aimed at preserving white matter integrity or modulating these neurotransmitter pathways requires further validation. Longitudinal research remains essential to determine whether these structural and molecular changes precede the clinical manifestation of dementia across the NIID spectrum.

## Data Availability

The original contributions presented in this study are included in this article/Supplementary material, further inquiries can be directed to the corresponding authors.

## Footnotes

1
http://rfmri.org/dpabi

2
http://www.nitrc.org/projects/panda

3
http://www.fmrib.ox.ac.uk/fsl

4
http://trackvis.org/dtk

5
http://www.fil.ion.ucl.ac.uk/spm
