# Supplementary material for: Structural and molecular substrates underlying functional dysconnectivity and cognitive impairment in neuronal intranuclear inclusion disease
Source: Front Behav Neurosci. 2026 Jul 3;20:1854155. doi: 10.3389/fnbeh.2026.1854155 (PMC13375799; doi:10.3389/fnbeh.2026.1854155)
Supplement: Supplementary file 1 [file Data_Sheet_1.docx]

**Supplementary tables**

**Table S1**. Post-hoc power analysis for white matter tracts (NIID vs. HC)

| Tract | HC mean ± SD | PT mean ± SD | *t* | *p* | Cohen's *d* | Power |
| --- | --- | --- | --- | --- | --- | --- |
| L_ATR_FA | 0.367±0.011 | 0.306±0.044 | 4.05 | 8.3e-4 | 1.86 | 96.8% |
| R_ATR_FA | 0.353±0.008 | 0.298±0.040 | 4.07 | 8.0e-4 | 1.87 | 96.9% |
| Forceps_minor_FA | 0.413±0.011 | 0.357±0.043 | 3.77 | 0.0015 | 1.73 | 94.4% |
| L_ATR_MD | 0.00114±0.00006 | 0.00148±0.00028 | -3.55 | 0.0025 | 1.63 | 91.7% |
| L_ATR_AD | 0.00065±0.00005 | 0.00101±0.00029 | -3.69 | 0.0018 | 1.70 | 93.5% |
| R_ATR_AD | 0.00066±0.00007 | 0.00098±0.00026 | -3.62 | 0.0021 | 1.67 | 92.7% |
| L_ATR_RD | 0.00081±0.00005 | 0.00116±0.00028 | -3.65 | 0.0020 | 1.68 | 93.0% |
| R_ATR_RD | 0.00081±0.00008 | 0.00113±0.00026 | -3.56 | 0.0024 | 1.63 | 91.8% |

**Table S2**. Correlation analyses between functional connectivity, white matter integrity and cognitive performance in NIID patients.

| **Variable 1** | **Variable 2** | ***r*_Pearson** | ***p*_raw_Pearson** | ***p*_FDR_Pearson** | ***r*ho_Spearman** | ***p*_raw_Spearman** | ***p*_FDR_Spearman** | **BCa_2.5%** | **BCa_97.5%** | **Robust** |
| --- | --- | --- | --- | --- | --- | --- | --- | --- | --- | --- |
| L_ATR _FA | MOCA | 0.6395 | 0.0636 | 0.1273 | 0.6193 | 0.0753 | 0.1627 | -0.3183 | 0.9487 | No |
| L_ATR _FA | FCS in R_IFGoperc | 0.7191 | **0.029** | 0.1258 | 0.75 | **0.0199** | 0.1296 | 0.4087 | 0.9411 | **Yes** |
| L_ATR _FA | FCS in L_REC | 0.3701 | 0.3269 | 0.3541 | 0.477 | 0.1942 | 0.2781 | -0.5897 | 0.8621 | No |
| R_ATR_FA | MOCA | 0.5791 | 0.1023 | 0.1772 | 0.4686 | 0.2032 | 0.2781 | -0.5435 | 0.8919 | No |
| R_ATR_FA | FCS in R_IFGoperc | 0.6281 | 0.0701 | 0.1302 | 0.6167 | 0.0769 | 0.1627 | -0.2342 | 0.8957 | No |
| R_ATR_FA | FCS in L_REC | 0.3748 | 0.3203 | 0.3541 | 0.41 | 0.273 | 0.2958 | -0.5687 | 0.8462 | No |
| Forceps_minor_FA | MOCA | 0.7656 | **0.0162** | 0.104 | 0.6862 | **0.0412** | 0.1627 | -0.0794 | 0.9483 | No |
| Forceps_minor_FA | FCS in R_IFGoperc | 0.6836 | **0.0423** | 0.127 | 0.6667 | **0.0499** | 0.1627 | -0.1927 | 0.9487 | No |
| Forceps_minor_FA | FCS in L_REC | 0.2252 | 0.5602 | 0.5826 | -0.1172 | 0.764 | 0.7946 | -0.7032 | 0.8714 | No |
| L_ATR_MD | MOCA | -0.6489 | 0.0586 | 0.127 | -0.6025 | 0.086 | 0.1627 | -0.8978 | 0.3378 | No |
| L_ATR_MD | FCS in R_IFGoperc | -0.7876 | **0.0117** | 0.104 | -0.7667 | **0.0159** | 0.1296 | -0.9813 | -0.2532 | **Yes** |
| L_ATR_MD | FCS in L_REC | -0.398 | 0.2888 | 0.3541 | -0.41 | 0.273 | 0.2958 | -0.8438 | 0.5897 | No |
| L_ATR_AD | MOCA | -0.649 | 0.0586 | 0.127 | -0.6025 | 0.086 | 0.1627 | -0.8978 | 0.3378 | No |
| L_ATR_AD | FCS in R_IFGoperc | -0.7781 | **0.0135** | 0.104 | -0.7667 | **0.0159** | 0.1296 | -0.9813 | -0.2532 | **Yes** |
| L_ATR_AD | FCS in L_REC | -0.3775 | 0.3165 | 0.3541 | -0.41 | 0.273 | 0.2958 | -0.8438 | 0.5897 | No |
| R_ATR_AD | MOCA | -0.5621 | 0.1152 | 0.1784 | -0.4686 | 0.2032 | 0.2781 | -0.8919 | 0.5435 | No |
| R_ATR_AD | FCS in R_IFGoperc | -0.6502 | 0.058 | 0.127 | -0.6167 | 0.0769 | 0.1627 | -0.8957 | 0.2342 | No |
| R_ATR_AD | FCS in L_REC | -0.4392 | 0.2369 | 0.3242 | -0.41 | 0.273 | 0.2958 | -0.8462 | 0.5687 | No |
| L_ATR_RD | MOCA | -0.6495 | 0.0583 | 0.127 | -0.6025 | 0.086 | 0.1627 | -0.8978 | 0.3378 | No |
| L_ATR_RD | FCS in R_IFGoperc | -0.7821 | **0.0128** | 0.104 | -0.7667 | **0.0159** | 0.1296 | -0.9813 | -0.2532 | **Yes** |
| L_ATR_RD | FCS in L_REC | -0.384 | 0.3076 | 0.3541 | -0.41 | 0.273 | 0.2958 | -0.8438 | 0.5897 | No |
| R_ATR_RD | MOCA | -0.5603 | 0.1166 | 0.1784 | -0.4854 | 0.1854 | 0.2781 | -0.9483 | 0.5514 | No |
| R_ATR_RD | FCS in R_IFGoperc | -0.65 | 0.0581 | 0.127 | -0.6 | 0.0876 | 0.1627 | -0.8783 | 0.0704 | No |
| R_ATR_RD | FCS in L_REC | -0.4524 | 0.2215 | 0.3199 | -0.477 | 0.1942 | 0.2781 | -0.8609 | 0.6316 | No |
| FCS in R_IFGoperc | MOCA | 0.7497 | **0.02** | 0.104 | 0.7113 | **0.0317** | 0.1627 | -0.2383 | 0.9954 | No |
| FCS in L_REC | MOCA | -0.0654 | 0.8672 | 0.8672 | 0.0168 | 0.9658 | 0.9658 | -0.7937 | 0.8869 | No |

**Notes:** FCS, functional connectivity strength; FA, fractional anisotropy; MD, mean diffusivity; AD, axial diffusivity; RD, radial diffusivity; ATR, anterior thalamic radiation; IFGoperc, inferior frontal gyrus, opercular part; REC, gyrus rectus; MoCA, Montreal Cognitive Assessment; BCa CI, bias-corrected and accelerated bootstrap confidence interval. Robustness was defined as the 95% BCa CI not encompassing zero. p_FDR was calculated using the Benjamini-Hochberg procedure.
